# Sexual Dimorphism of the Neuroimmunoendocrine Response in the Spleen during a Helminth Infection: A New Role for an Old Player?

**DOI:** 10.3390/pathogens11030308

**Published:** 2022-03-01

**Authors:** Karen Elizabeth Nava-Castro, Lenin Pavón, Luis Enrique Becerril-Villanueva, María Dolores Ponce-Regalado, Hugo Aguilar-Díaz, Mariana Segovia-Mendoza, Jorge Morales-Montor

**Affiliations:** 1 Laboratorio de Biología y Química Atmosférica, Departamento de Ciencias Ambientales, Instituto de Ciencias de la Atmósfera y Cambio Climático, Universidad Nacional Autónoma de México, Ciudad de Mexico 04510, Mexico; karlenc@atmosfera.unam.mx; 2 Laboratory of Psychoimmunology, National Institute of Psychiatry “Ramón de la Fuente Muñiz”, Mexico City 14370, Mexico; lkuriaki@imp.edu.mx (L.P.); lusenbeve@imp.edu.mx (L.E.B.-V.); 3 Centro Universitario de los Altos, Departamento de Ciencias de la Salud, Universidad de Guadalajara, Jalisco 47610, Mexico; dorispr@udg.edu.mx; 4 Centro Nacional de Investigaciones Disciplinarias en Salud Animal e Inocuidad, Instituto Nacional de Investigaciones Forestales Agrícolas y Pecuarias (INIFAP), Morelos 50550, Mexico; hugoaguilar@ciencias.unam.mx; 5 Departamento de Farmacología, Facultad de Medicina, Universidad Nacional Autónoma de México, Ciudad de Mexico 00810, Mexico; mariana.segovia@facmed.unam.mx; 6 Departamento de Inmunología, Instituto de Investigaciones Biomédicas, Universidad Nacional Autónoma de México, Ciudad de Mexico 04510, Mexico

**Keywords:** neuroimmunoendocrinology, spleen, parasite immunity, sexual dimorphism, neurotransmitters, cytokines, Helminths, cysticercosis, *Taenia crassiceps*, immunity, infection

## Abstract

The interaction of the nervous, immune, and endocrine systems is crucial in maintaining homeostasis in vertebrates, and vital in mammals. The spleen is a key organ that regulates the neuroimmunoendocrine system. The *Taenia crassiceps* mouse system is an excellent experimental model to study the complex host–parasite relationship, particularly sex-associated susceptibility to infection. The present study aimed to determine the changes in neurotransmitters, cytokines, sex steroids, and sex-steroid receptors in the spleen of cysticercus-infected male and female mice and whole parasite counts. We found that parasite load was higher in females in comparison to male mice. The levels of the neurotransmitter epinephrine were significantly decreased in infected male animals. The expression of IL-2 and IL-4 in the spleen was markedly increased in infected mice; however, the expression of Interleukin (IL)-10 and interferon (IFN)-γ decreased. We also observed sex-associated differences between non-infected and infected mice. Interestingly, the data show that estradiol levels increased in infected males but decreased in females. Our studies provide evidence that infection leads to changes in neuroimmunoendocrine molecules in the spleen, and these changes are dimorphic and impact the establishment, growth, and reproduction of *T. crassiceps*. Our findings support the critical role of the neuroimmunoendocrine network in determining sex-associated susceptibility to the helminth parasite.

## 1. Introduction

Murine intraperitoneal cysticercosis is caused by the helminth cestode *Taenia crassiceps.* It has been useful to explore the host factors associated with porcine cysticercosis and, to some degree, with human neurocysticercosis. Intraperitoneal *T. crassiceps* cysticercosis of mice lends itself well to controlled and reproducible experimentation, generating substantial parasite loads in individual mice in a matter of a few weeks after infection [1,2]. This experimental model allows for a good representation of other forms of cysticercosis because of the parasite’s extensive sharing of antigens with other taenias and cestodes, since *T. crassiceps* and *T. solium* have high genetic homology [3,4,5,6]. These characteristics have made murine cysticercosis a convenient instrument for testing vaccine candidates and new treatments against cysticercosis and evaluating the complex host-cysticercus interaction [7,8]. Several features of *T. solium* cysticercosis disease have been found by extrapolation from experimental murine cysticercosis [9,10]. Particularly, experimental murine *T. crassiceps* cysticercosis has revealed the complexities of the interactive network that regulates infection, formed by the immune and neuroendocrine systems of the host and the parasite [9]. In *T. crassiceps* cysticercosis, females of all the strains of mice that were studied sustain greater intensities of infection than males. However, during chronic infection (more than 4 weeks), this difference tends to disappear, and the males of the BALB/c strain show a feminization process, characterized by high serum estrogen levels (200 times the normal values) whilst testosterone is 90% decreased [11,12]. At the same time, the cellular immune response (Th1) is markedly diminished in both sexes, and the humoral (Th2) response is enhanced [13]. In addition, estradiol is involved in the immunoendocrine regulation of murine *T. crassiceps* cysticercosis as a major factor promoting cysticercus growth, through deregulating the thymus-dependent cellular mechanisms that obstruct parasite growth [14]. Furthermore, gonadectomy, which decreases the serum levels to an undetectable level, alters the growth of the parasites, giving similar parasitic loads in both sexes [15]. Interestingly, the absence of estrogens does not prevent the growth of the parasites in both genders, demonstrating that although estradiol favors *T. crassiceps* development, it is not indispensable for rapid parasite growth [16].

Sexual dimorphism (SD) in infections due to parasites is a scarcely studied biological phenomenon of considerable significance for individual health, behavior, and lifestyles, as well as for the evolution of species [11,17]. Particularly in the hosts, cysticercosis has been more prevalent in females, which carry larger parasite loads, more severe infection, and higher resistance to develop protective immunity to variable degrees. These differences are associated with their genetic background and the duration of the infection [18]. Mechanisms underlying sexual dimorphism in murine cysticercosis consist of a complex network. The host’s prominent sex steroids and their receptors, along with other effectors in the immune and central nervous systems, interact with each other and with the parasite [19].

For several years, our workgroup has been studying the neuroimmunoendocrine control of *T. crassiceps* infection [18,20,21]. However, neuroimmunoendocrine modulation of *T. crassiceps* infection in the spleen has never been studied in both sexes. The coordination of the immune system is essential for physiological homeostasis and critical for the proper response to infection [1,22,23,24,25]. At the same time, the unregulated production of molecules from the neuroimmunoendocrine network is one of the principal causes of morbidity and mortality [26,27,28]. As part of the neuroimmunoendocrine network, the autonomic nervous system connects the immune and endocrine system with the other organs and orchestrates the immune responses according to physiological needs [29]. Sympathetic and parasympathetic systems oppose maintaining physiological homeostasis [30]. Although the sympathetic system has been studied for years, the anti-inflammatory potential of the parasympathetic system was just recently demonstrated [31,32]. Nevertheless, there is not much evidence about its participation during the infection by *T. crassiceps*.

This study aimed to investigate neuroimmunoendocrine changes in the spleen of male and female infected mice with *T. crassiceps* and to correlate these data with the parasite burdens. Our results provide evidence that chronic infection leads to persistent changes in neuroimmunoendocrine function in the spleen. These findings support the crucial role of neuroimmunoendocrine network in sexual dimorphism under *T. crassiceps* infection.

## 2. Results

### 2.1. Number of Parasites

**Figure 1:**
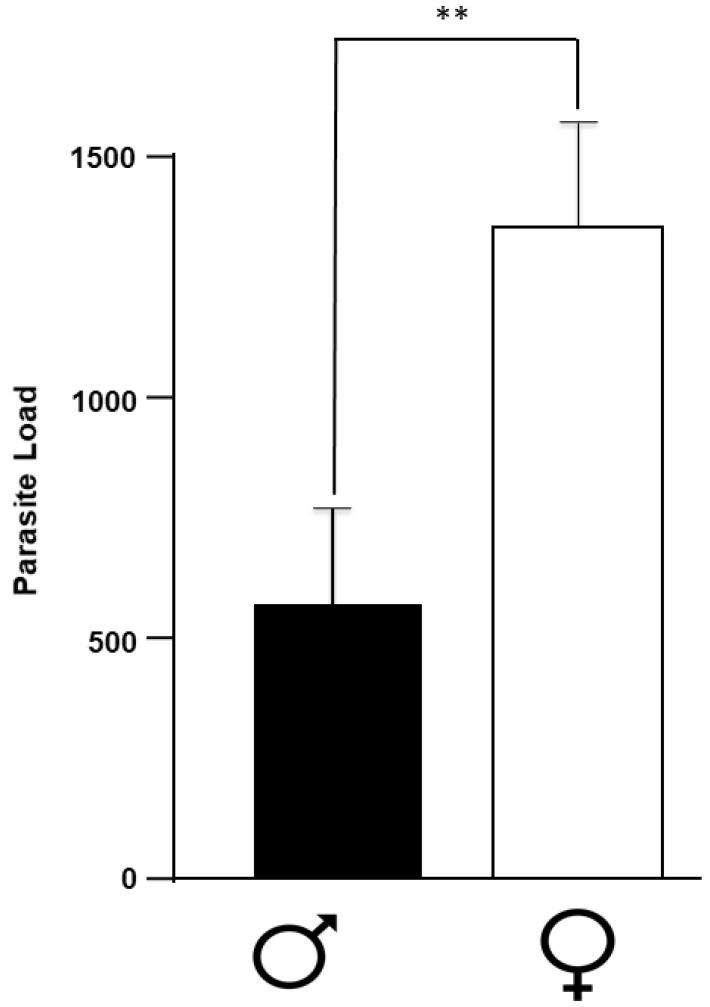
Parasite load in infected mice of both sexes. Data show the average of the number of parasites recovered from the peritoneal cavity of 10 female (white bar) and 10 male (black bar) BALB/c mice at 16 weeks post-infection. Each bar represents the average +/− SD in every sex at 16 weeks post-infection. **, *p* < 0.001 when comparing male and female infected mice.

Figure 1 shows the individual number of parasites recovered from infected mice of both sexes. As expected, we observed an evident sex-associated susceptibility to the infection. Female mice reached a parasite load of 1346.6 ± 189.6 per mouse at 16 weeks of infection, while male mice showed 2.33 less fold the parasite burden than females (576.6 ± 166.0) (Figure 1). Parasites were found exclusively in the peritoneal cavity of infected mice, and never in any other organ or compartment of the host. Also, mice did not have any apparent sign of disease. The infected mice did not lose weight, and their fur’s appearance was maintained throughout the experiment.

### 2.2. Levels of Neurotransmitters in the Spleen

**Figure 2:**
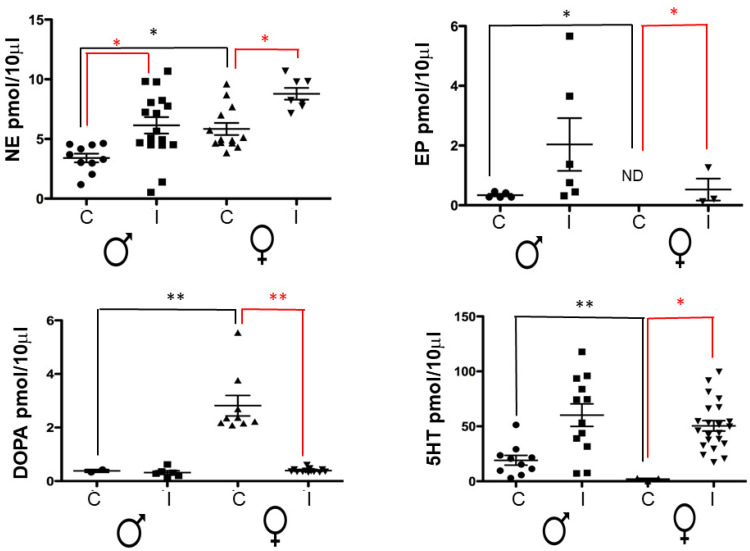
Splenic levels of neurotransmitters in the control groups and in female and male mice infected with *T. crassiceps*. The levels of NE, EP, DOPA, and 5HT were measured in the spleen of the different experimental groups. Data show the levels of neurotransmitters corresponding to 20 female (10 controls and 10 infected) and 20 male (10 controls and 10 infected) BALB/c mice at 16 weeks post-infection The black lines show differences between gender, the red lines show differences between control and infected animals. *p* < 0.05 *, *p* < 0.01 **. Abbreviations: ND, Not Detected, NE, norepinephrine; EP, epinephrine; DOPA, dopamine; 5-HT, serotonin; C, uninfected control; I, infected. Significance in black indicates difference between the control groups of both sexes. Significance in red color indicates difference between the control group and the infected group of the same sex.

Figure 2 shows the splenic concentrations of four neurotransmitters in infected mice of both sexes. As depicted, neurotransmitter levels in infected groups are higher than those found in controls except for dopamine (DOPA), which was higher in controls of female mice. The levels of norepinephrine (NE), epinephrine (EP), and serotonin (5-HT) were sexually dimorphic. NE was significantly higher in infected female mice when compared to their male counterparts and controls (Figure 2). In contrast, the levels of EP were greater in infected male mice, while in the infected females, the concentration of this neurotransmitter was minimal. Finally, the levels of 5-HT were similar in both male and female infected mice; however, the uninfected males showed significantly superior levels of this neurotransmitter than the uninfected females. In summary, the changes in neurotransmitter content in the spleen of the infected groups were different from those in the non-infected groups.

### 2.3. IL-2, IL-4, and IL-10 Spleen Expression

**Figure 3:**
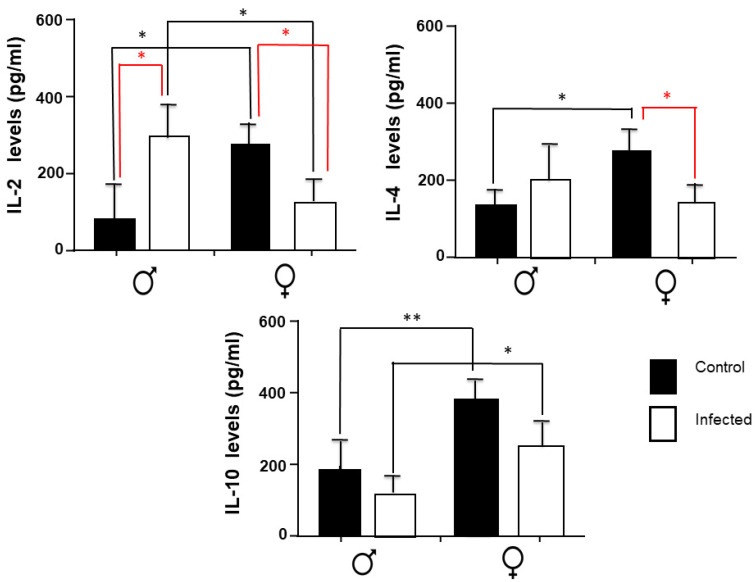
Levels of splenic IL-2, IL-4, and IL-10 in the control groups and in male and female mice infected with *T. crassiseps*. Data show the levels of different cytokines corresponding to 20 female (10 controls and 10 infected) and 20 male (10 controls and 10 infected) BALB/c mice at 16 weeks. The black lines show differences between gender; the red lines show differences between control and infected animals. *p* < 0.05 *, *p* < 0.01 **. Significance in black indicates difference between the control groups of both sexes. Significance in red color indicates difference between the control group and the infected group of the same sex.

The levels of IL-2, IL-4, and IL-10 in the spleen obtained from male and female mice in response to *T. crassiseps* infection are shown in Figure 3. IL-2 levels significantly increased in the infected group of males with respect to the control. In contrast, the infected females exhibit a significant decrease in IL-2 levels compared to the control group Interestingly, IL-4 significantly decreased only in infected females compared to the control group. No significant changes were observed between the infected groups and their control for IL-10. In control groups, we found that females have higher IL-2 and IL-4 compared to males.

### 2.4. IFN-γ, TNF-α, and IL-6 Expression in the Spleen

**Figure 4:**
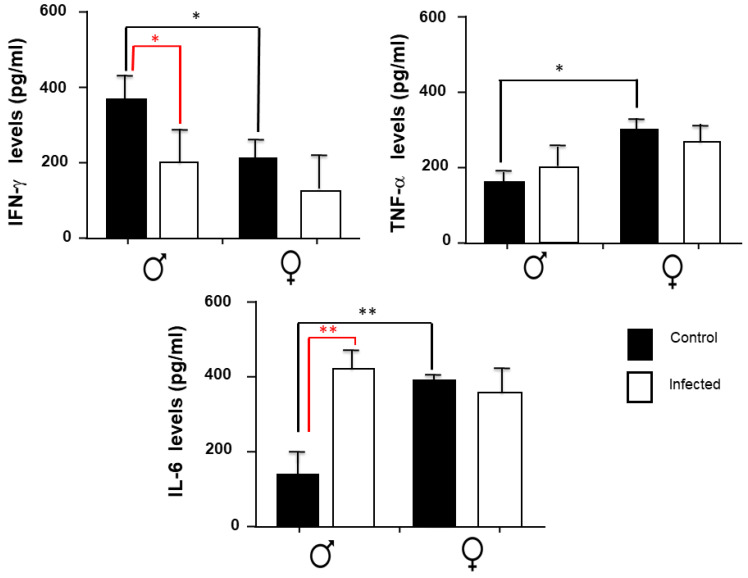
Levels of splenic IFNγ, TNF-α, and IL-6 in the control groups and in male and female mice infected with *T. crassiseps*. Data show the levels of different cytokines corresponding to 20 female (10 controls and 10 infected) and 20 male (10 controls and 10 infected) BALB/c mice at 16 weeks. The black lines show differences between gender; the red lines show differences between control and infected animals. *p* < 0.05 *, *p* < 0.01 **. Significance in black indicates difference between the control groups of both sexes. Significance in red color indicates difference between the control group and the infected group of the same sex.

In Figure 4, it is depicted the expression of IFN-γ, TNF-α, and IL-6 expression in the spleen. IFN-γ is decreased in infected males; however, females do not have differences between groups. For TNF-α, no differences were observed between the sexes in the infected and control groups. In the case of IL-6, infected males had an increase compared to the control group. Nonetheless, females did not have differences between groups.

Sexual dimorphism is also observed in control groups for IFN-y, in which males have higher levels. Contrary, control females exhibit higher levels of TNF-α and IL-6 compared to the control group of males. In general terms, *T. crassiseps* infection causes alterations of these basal conditions in IL-6 levels. Concerning female mice, infection by *T. crassiseps* also reduced their basal profile, decreasing the levels of IFN-γ. The infection did not modify the levels of TNFα to this parasite.

### 2.5. Sex Steroids Levels in the Spleen

**Figure 5:**
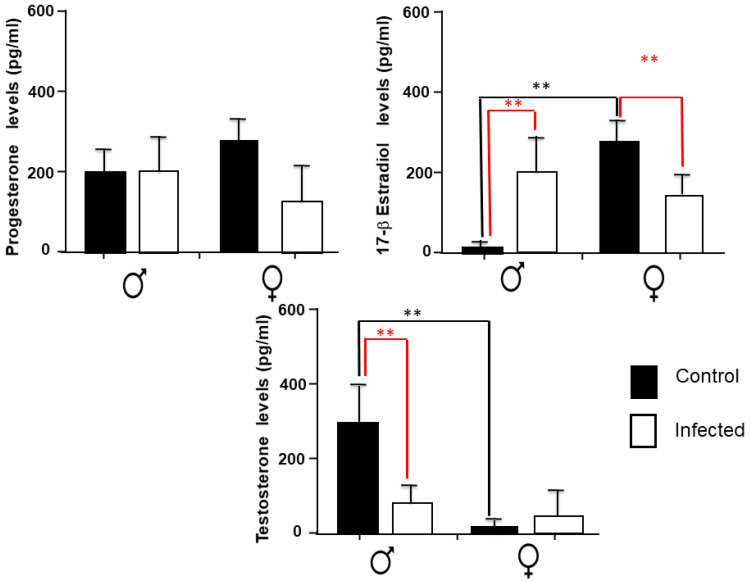
Splenic levels of steroid hormones, progesterone, estradiol, and testosterone, in the control groups and in male and female mice infected with *T. crassiseps*. Data show the levels of different hormones corresponding to 20 female (10 controls and 10 infected) and 20 male (10 controls and 10 infected) BALB/c mice at 16 weeks post-infection, *p* < 0.001 ** when comparing the control group and infected individuals. The black lines show differences between gender, the red lines show differences between control and infected animals. *p* < 0.01 **. Significance in black indicates difference between the control groups of both sexes. Significance in red color indicates difference between the control group and the infected group of the same sex.

We measured the splenic content of progesterone, estradiol, and testosterone in all groups (Figure 5). Progesterone levels were not modified due to the infection in both sexes (Figure 5). In the case of estradiol, as expected, an evident sexual dimorphism was observed in control females, the group with higher levels of this hormone than males. However, the infection with *T. crassiseps* markedly increased estradiol levels in male animals, evoking similar levels to those of control females. Conversely, females significantly reduced estradiol content in the spleen (Figure 5). Testosterone levels were also dimorphic, the males now being the group that had the higher levels. However, as in estradiol, testosterone levels were altered in the infected male and female population; males had a significant decrement in the production of this hormone; meanwhile, infected females had a slight increase compared to the control group (Figure 5).

### 2.6. Expression of Sex Steroid Receptors in the Spleen

**Figure 6:**
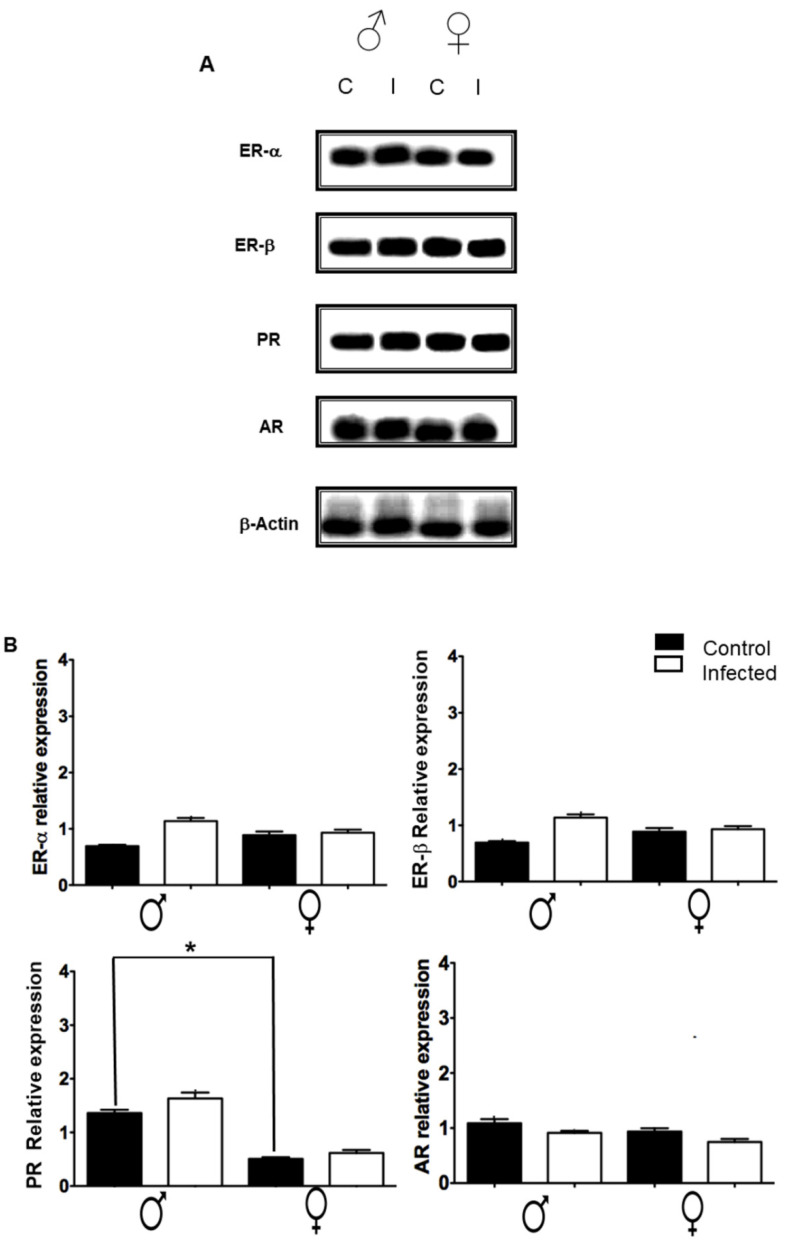
Splenic levels of the different steroid receptors, ERα, ERβ, PR, and AR, evaluated in male and female mice non-infected and infected with *T. crassiseps*. A representative image (**A**) and the densitometry analysis of the different experiments (**B**) are shown. β-actin was used as the loading control. Normalization of values was carried out against the loading control. 20 female (10 controls and 10 infected) and 20 male (10 controls and 10 infected) BALB/c mice at 16 weeks were used. The black lines show differences between gender. *p* < 0.05 *.

The expression of nuclear estrogen receptors (ERα and Erβ), progesterone receptor (PR), and androgen receptor (AR) in the spleen of the mice were measured by Western blot (Figure 6). Interestingly, the infection of *T. crassiseps* did not induce significant changes in the expression of ERα, Erβ, or AR in the infected male and female mice compared with their control group. Additionally, PR was the only receptor that showed a significant dimorphic effect in animals without infection; however, the infection did not affect its tendency in any infected counterpart.

Finally, we analyze the correlation of all parameters with sex, infection, and both together. As expected, our analysis showed a positive correlation (r = 0.95) between sex and parasite loads. As for sex and neurotransmitter levels, there was a positive correlation (r = 0.90), since dopamine increases in non-infected females, while epinephrine and serotonin increased in infected females.

Cytokine levels have also a positive correlation between sex the levels of IFNγ were higher in control males, meanwhile, TNF-α and IL-6 were higher in females without infection, indicating that females have a basal proinflammatory profile. In the case of IFNγ in males, infection by *T. crassiceps* induced a decrease in this cytokine. TNFα remained unchanged in animals with or without infection. And IL6 was only elevated in infected males.

In the case of sex steroids levels, as expected, only 17-β estradiol and testosterone positively correlate with sex (r = 0.92). Males have less estradiol and higher testosterone levels than control females, and there was no correlation between sex and sex steroid receptor expression. As for infection, the stronger positive correlation was between neurotransmitter and cytokine levels.

## 3. Discussion

Few studies have investigated the effects of chronic infection on the neuroimmunoendocrine response in lymphoid organs [28]. The spleen is the largest secondary immune organ. It comprises two main compartments: (1) the red pulp, which filters the blood of foreign material and damaged erythrocytes, and (2) the white pulp, which initiates immune reactions to blood-borne antigens [31]. To our knowledge, this is the first study to demonstrate a relationship between helminth infection and the neuroimmunoendocrinology of the spleen. Additionally, we provide evidence that this interaction depends on the sex of the individual.

The interaction of the nervous, endocrine, and immune systems is crucial in maintaining homeostasis in vertebrates, and it is vital in mammals [32,33]. The immune system’s capacity to discriminate between self and non-self is based on the high specificity poised by immune cells. The immune response plays a key role in controlling physiology, contributing to maintaining the integrity of the body cells and tissues antigens [34]. Hormones and neurotransmitters present in the immune cell microenvironment can restrict their autonomy, probably by acting through their receptors located on immune cells. Efficient communication among these three systems implies the existence of afferent and efferent pathways, constituting a complex feedback system, which, when altered, can trigger different pathologies [35,36].

Since this work aimed to study these neuroimmunoendocrine interactions, we evaluated the biological components of these three systems, that is, we measured cytokines as indicators of immune function, neurotransmitters as components of the neuronal axis, and hormones that are involved in the endocrine network. We observed that infected male and female mice had a significant increase of NE and 5-HT in the spleen (Figure 2). In addition, we found a dimorphic response to the infection since EP was higher in infected males than in infected females.

Serotonin levels have also been reported to increase when an infection occurs; this increase is associated with eradicating the disease via multiple pathways [37,38]. We also observed a significant increase in NE in both sexes, which other studies have associated with Th2 polarization and M2 macrophage differentiation as well as an increase in nitric oxide (NO) production) [39,40,41]. In addition, the increase in 5-HT is related to T and B lymphocyte proliferation, which enhances the establishment of a humoral response to infection [42,43].

Cytokines are other soluble factors that are secreted in response to infections [44,45,46]. We explored the two counterparts involved in inflammation regarding the immune system, T helper type 1 (TH1) and T helper type 2 (TH2). Usually, mammals employ the T helper type 2 (TH2) immune response to protect against helminth infections [47,48]. Related to this, we observed that IL-6 was upregulated in healthy and infected female mice. Different reports have demonstrated that the deficiency of this cytokine is related to the absence of parasitic burden, increased immunity, and resistance to the parasite in an *H. polygyrus* infection mouse model.

Moreover, IL-6 can modulate different lineages of T lymphocytes and antibody production [49]. In contrast, the upregulation of this cytokine has opposite effects. According to the above, the results presented here agree with this assumption because elevated levels of IL-6 correlate with higher parasitic loads in females compared to males. Our results also follow previous reports that mention that IL-4 is increased after intestinal nematode infection [42]. We were only able to corroborate these findings in infected males. On the other hand, Infected females showed a significant decrease compared to their control group. The preceding suggests that the infection with T. crassiceps is dimorphic, which could be accompanied by the modulation of different immune lineages that need further study.

Interestingly, we observed an increase in estradiol levels in infected males, while females decreased this hormone. These changes were not accompanied by differences in the expression of its nuclear receptors. Additionally, the testosterone levels in male mice were also negatively affected, in a similar manner to its corresponding receptor. This is in accordance with other studies that report that intracellular protozoan parasite infections can impact sex hormones and the immune response [50,51]. Therefore, our data also support the existing literature regarding sexual dimorphism in the susceptibility to parasitic diseases.

Moreover, it is known that helminth infections activate the Th2 response, while protozoan infections can trigger the Th1 response [52,53,54]. In our model, infection by T. crassiceps appears to mimic helminth infections caused by Fasciola hepatica and Schistosoma mansoni, polarizing the response to a Th2 phenotype instead of favoring a Th1, with its respective increase in IFN-γ [52,53,54]. We related this increased response to the high levels of IL-6 observed mainly in male mice. On the other hand, the increase in IL-6 could even explain the elevation in estradiol levels in male mice, since extensive literature relates this cytokine to the induction of aromatase, the enzyme responsible for the synthesis of estrogens from androgens [55].

The findings presented in this paper must be interpreted considering some limitations. First, our design does not allow for causal inferences. Past experimental evidence suggests that host-parasite interactions are associated with changes in neural activity, interleukin and cytokine production, and hormones in the host, which interact with the parasites’ survival and reproduction [20,21,25,56]. The most important feature of these changes is that they are dimorphic, so the response of each sex determines parasite adaptation and growth.

The possibility that the parasympathetic innervation to the spleen directly senses peritoneal components through its nerve endings cannot be excluded. Considering that: (1) intraperitoneal murine cysticercosis is accompanied by a number of unique events (i.e., a bias towards the female environment, feminization of male hosts, a permissive TH2-inclined immune response) [11,57], (2) this infection does not kill the host despite the huge parasite loads, and (3) the host’s immune response does not reject the parasite, one is led to suspect that such host-parasite conviviality rests upon unusually effective mechanisms that enable physiological stability for both host and parasite, whereby the spleen it is a key player.

Finally, our results indicate that parasite stimuli can influence cellular events within the spleen, which is considered one of the most specific peripheral organs of the immune response, and suggest that a distinctive feature of the infection is the change in neurotransmitters, cytokines, and hormones, which are involved with the control of parasite growth in a sexually dimorphic fashion. Regardless of how they arise, the changes observed in the spleen of the infected male and female mice may be something more than just an interesting observation: they may also indicate a general host reaction and the involvement of the host-parasite relationship in acquired and innate immune responses, as well as behaviors. Thus, parasite infections can induce systemic alterations such as malnutrition, anemia, and impaired cognitive functions [58,59], which should be considered in a preponderant way.

## 4. Materials and Methods

### 4.1. Ethics Statement

Animal care and experimental practices were conducted at the Unidad de Modelos Biológicos (UMB) at the Instituto de Investigaciones Biomédicas (IIB), Universidad Nacional Autónoma de Mexico. All experimental procedures were approved by the Institutional Care and Animal Use Committee (CICUAL, permit number 2015-00134, Mexico City) adhering to the Mexican regulation (NOM-062-ZOO-1999, Mexico City), and in accordance with recommendations from the National Institute of Health (NIH) of the United States of America (Guide for the Care and Use of Laboratory Animals). The euthanasia of experimental animals was performed by an overdose of sevoflurane.

### 4.2. Animals and Experimental Infections

Male and female Balb/c AnN (H2-d) inbred mice obtained from Harlan (Mexico City, Mexico) were used in all experiments. Animals were reproduced and housed in the animal care facilities at Instituto de Investigaciones Biomédicas, (UNAM), under controlled temperature and a 12 h dark-light cycle with lights switched on between 0700 and 1900. They were fed Purina Diet 5015 (Purina, St. Louis, MO, USA) and given tap water ad libitum. Puberty onset—vaginal opening—as well as the immediate cyclicity, were evaluated in females. In all experiments, the fast-growing ORF strain of *T. crassiceps* was used for infection [60]. Larvae were obtained from infected 3–6-month female donor mice. Ten non-budding *T. crassiceps* larvae (approximately 2 mm in diameter) were suspended in 0.3 mL sterile phosphate-buffered saline (PBS: 0.15M NaCl, 0.01M sodium phosphate buffer, pH 7.2) and intraperitoneally injected into each of eight to ten 42-day-old male and female mice using a 0.25-gauge needle. Ten non-infected mice of each sex were used as age-matched controls. Mice were rapidly euthanized by an overdose of sevoflurane (Pfizer, Mexico) at 16 weeks of infection. Vaginal smears were previously taken from females to monitor the estrous cycle; euthanasia was performed in metaestrous. Peritoneal cysticerci were collected and counted after rinsing the peritoneal cavity with PBS. Spleens were collected immediately after rinsing.

### 4.3. Tissues Sample

The spleen of infected and normal mice of both sexes was excised immediately after euthanasia and processed as described. After euthanasia, mice spleens were rapidly removed and chilled in cold 0.9% saline solution. All spleens were split into three parts, that were processed according to the variable to be measured (nervous, immune, or endocrine).

### 4.4. Neurotransmitter Measurement

Fresh spleens (a third of the total tissue) were placed in a 1.5 mL microcentrifuge tube on dry ice. Ice-chilled 0.1 M PCA containing internal standard (∼10 mg tissue in 300 μL PCA) was added into the tissue tube. Then, the tubes were placed on crushed ice or at 4 °C, until the PCA thawed. Samples were sonicated briefly with a microprobe fitted in the sample tube (6–7 s, duty cycle 80%, output control 3) until the tissue was completely homogenized. The tubes were kept on crushed ice or at 4 °C for 10 min. After a short vortex of samples, 30 μL of homogenate were set apart for the protein assay. Samples were then centrifuged at 14,000 rpm (18,000× *g*) for 15 min, at 4 °C. The supernatant was transferred into another clean 1.5 mL microcentrifuge tube, which was the supernatant was used for HPLC assay to determine neurotransmitters (serotonin, dopamine, and other monoamines). The system, in sequence, consists of a pump, a pulse damper, an autosampler, a PEEK in-line filter, a column, a conditioning cell, and analytical cells. The fresh mobile phase was passed through the system overnight in a recycling mode before HPLC analysis. The system parameters were as follows: flow rate: 1.0 mL/min, column oven temperature: 30 °C, injection volume: 15 μL, autosampler sample chamber temperature: 4 °C, applied potentials (mV): conditioning cell = +10; analytical cell, E1  =  +50; E2  =  +340. Once the system was set up and equilibrated, the concentration series of the standard mixture of monoamines and their metabolites were assessed, to make sure a clear chromatographic separation was obtained. Also, the individual standards to identify each peak in the mixture by its retention time and voltammetric response were carried out. Readings of peak height and peak area were obtained by using the analytical system software. Sensitivity was determined by cutting off the ratio of the signal peak when the noise peak was lower than 5 for every monoamine or metabolite. An internal standard was used to plot the standard curve of each monoamine and metabolite and their least-squares linear regression equations were obtained using Microsoft Office Excel. Concentrations of monoamines and their metabolites in the sample were calculated by using standard equations. All the monoamines and their metabolites in samples were well eluted in <18 min; and afterward, the baseline did not show more interfering peaks. The order and retention time of the eluted monoamines and their metabolites as seen in the chromatogram were: NE (2.38 min), DOPAC (4.17 min), DA (5.29 min), 2,3-DHBA (6.99 min), 5-HIAA (7.61 min), HVA (9.83 min), and 5-HT (14.9 min).

### 4.5. Cytokine Spleen Expression

Spleen cytokines were measured with the ABTS ELISA kit (Cat# 900-K00). According to the manufacturer’s instructions, antibodies for IL-2, IL-4, IL-6, IL-10, TNF-α, IFN-γ, and unconjugated antibodies were used for cytokine capture, with a few modifications. Briefly, coated plates (96-well plate, MaxiSorp Nunc Cat. NNC#442404) were incubated with 50 µL (2 µg/mL) of different antibodies overnight. After rinsing three times, (Wash buffer, PeproTech, East Windsor, NY, USA), the plates were blocked (Block buffer, PeproTech) and then washed again. Then, 50 µL of spleen protein (10 µg) were added to the plate in duplicates (diluent solution, PeproTech). The samples were maintained at 4 °C for 2 h and washed three times. An enzyme-substrate reaction was developed with ABTS liquid substrate (PeproTech). All solutions were obtained from the ABTS ELISA buffer kit. The plates were read at a wavelength of 405 nm with a wavelength correction set at 650 nm at different time points in a Stat Fax 4200 microplate reader (Awareness Technology, Palm City, FL, USA).

### 4.6. Sex Steroids Levels in the Spleen

Freshly extracted spleens were homogenized in PBS. Steroids were ether-extracted as previously reported [13] and solubilized in the phosphate buffer used for Enzyme-immunoassay (EIA). According to the manufacturer’s protocol, estradiol, testosterone, and dihydrotestosterone levels were detected using EIA DetectX^®^ kits (Arbor Assays, Ann Arbor, MI, USA).

### 4.7. Expression of Steroids Receptors (ER, PR, and AR) in the Spleen by Western Blot

In brief, as mentioned before, spleen proteins were extracted and disrupted in Tris–HCl (1 mL/0.1 g tissue) with a protease inhibitor cocktail (Calbiochem). The supernatant was recovered after 30 min of centrifugation at 12,000× *g*. The pellet was discarded. Protein concentration was obtained by absorbance at 580 nm using the Bradford–Lowry method. Total protein of every analyzed tissue (80 μg) was boiled in reducing Laemmli sample buffer, separated by SDS–PAGE (8% acrylamide) and electroblotted onto nitrocellulose membranes. Membranes were blocked for 1 h in SuperBlock T20 (Thermo Scientific, Waltham, MA, USA). For protein immunodetection in splenocytes, membranes were subjected to overnight immunoblotting with 1 μg/mL of polyclonal anti-ERα antibodies (HC-20, Santa Cruz Biotechnology, Dallas, TX, USA); ERb (Y-19, Santa Cruz Biotechnology); PR-A/PR-B (C-20, Santa Cruz Biotechnology); β-actin (HC20a-543, Merk/Millipore Sigma, Burlington, MA, USA). All antibodies were diluted 1:1000. After incubation with the primary antibody, samples were incubated with HRP-conjugated anti-mouse, anti-goat, or anti-rabbit IgG antibodies (Santa Cruz Biotechnology; diluted 1:10,000) for 1 h, at room temperature. Next, membranes were washed three times in TBST buffer (10 mM Tris–HCl, pH 7.4, 100 mM NaCl, 0.5% Tween 20) and the bands were visualized using the ECL system according to the manufacturer’s instructions (Super Signal ECL, Pierce, Invitrogen, Thermo Fisher Scientific, Waltham, MA, USA). Chemiluminescent signals were captured in Kodak Bio-Max Gel-Doc system film, and the bands were quantitatively analyzed from the digitized image using the Bio-Rad Quantity One software (Bio-Rad, Hercules, CA, USA). Mouse uterus was used as positive control of steroid receptor expression in this experiment. A single band was detected for ERα at 66 kDa, ERβ at 55 kDa and β-actin at 55 kDa, and two bands for PR at 110 kDa for PR-B and 85 kDa for PR-A. Sex steroid receptor expression level was normalized respective to β-actin.

### 4.8. Experimental Design and Statistical Analysis

We designed a two-factorial experiment. The independent variable was: (1) infection (two levels: Yes, No), (2) gender (two levels: male or female). The dependent variables were the number of parasites, the levels of each cytokine IL-2, IL-4, IL-6, L-10, IFN-γ, and TNF-α in the spleen, the levels of neurotransmitters, the levels of sex steroids, and expression of sex steroid receptors. The complete design was repeated twice, and the tissues used in each experiment at each time of infection were those from 10 normal or 10 infected mice (*n* = 20). Statistical analysis of multiple variance components was performed with Prism 2.01 (GraphPad Software, San Diego, CA, USA). When applicable, post hoc individual contrasts of group mean by the Tukey test used the sum of Residual and Three-Factor Interactions variance were performed to test for significant differences.

## Data Availability

Data may be requested from the corresponding author: jmontor66@iibiomedicas.unam.mx.

## References

[1] Larralde C., Sciutto E., Huerta L., Terrazas I., Fragoso G., Trueba L., Lemus D., Lomelí C., Tapia G., Montoya R.M. (1989). Experimental cysticercosis by *Taenia crassiceps* in mice: Factors involved in susceptibility. Acta Leiden.

[2] Larralde C., Sotelo J., Montoya R.M., Palencia G., Padilla A., Govezensky T., Diaz M.L., Sciutto E. (1990). Immunodiagnosis of human cysticercosis in cerebrospinal fluid. Antigens from murine *Taenia crassiceps* cysticerci effectively substitute those from porcine Taenia solium. Arch. Pathol. Lab. Med..

[3] Rishi A.K., McManus D.P. (1988). Molecular cloning of *Taenia solium* genomic DNA and characterization of taeniid cestodes by DNA analysis. Parasitology.

[4] Larralde C., Laclette J.P., Montoya R.M., Contreras L., Sandoval M., Bojalil R., Owen C.S., Arzate J., Goodsaid F., Diaz M.L. (1986). Reliable Serology of *Taenia solium* Cysticercosis with Antigens from Cyst Vesicular Fluid: Elisa and Hemagglutination Tests. Am. J. Trop. Med. Hyg..

[5] Toledo A., Fragoso G., Rosas G., Hernández M., Gevorkian G., López-Casillas F., Hernández B., Acero G., Huerta M., Larralde C. (2001). Two Epitopes Shared by *Taenia crassiceps* and Taenia solium Confer Protection against Murine *T. crassiceps* Cysticercosis along with a Prominent T1 Response. Infect. Immun..

[6] Landa A., Navarro L., Ochoa-Sánchez A., Jiménez L. (2019). *Taenia solium* and *Taenia crassiceps*: miRNomes of the larvae and effects of miR-10-5p and let-7-5p on murine peritoneal macrophages. Biosci. Rep..

[7] Sciutto E., Fragoso G., Manoutcharian K., Gevorkian G., Rosas-Salgado G., Hernández-Gonzalez M., Herrera-Estrella L.R., Cabrera-Ponce J.L., López-Casillas F., González-Bonilla C. (2002). New Approaches to Improve a Peptide Vaccine Against Porcine *Taenia solium* Cysticercosis. Arch. Med. Res..

[8] Sciutto E., Fragoso G., Trueba L., Lemus D., Montoya R., Diaz M.L., Govezensky T., Lomeli C., Tapia G., Larralde C. (1990). Cysticercosis vaccine: Cross protecting immunity with *T. solium* antigens against experimental murine *T. crassiceps* cysticercosis. Parasite Immunol..

[9] Morales J., Velasco T., Tovar V., Fragoso G., Fleury A., Beltrán C., Villalobos N., Aluja A., Rodarte L., Sciutto E. (2002). Castration and pregnancy of rural pigs significantly increase the prevalence of naturally acquired Taenia solium cysticercosis. Veter Parasitol..

[10] Sciutto E., Rosas G., Hernández M., Morales J., Cruz-Revilla C., Toledo A., Manoutcharian K., Gevorkian G., Blancas A., Acero G. (2007). Improvement of the synthetic tri-peptide vaccine (S3Pvac) against porcine *Taenia solium* cysticercosis in search of a more effective, inexpensive and manageable vaccine. Vaccine.

[11] Morales-Montor J., Chavarria A., De León-Nava M.A., Del Castillo L.I., Escobedo E.G., Sánchez E.N., Vargas J.A., Hernández-Flores M., Romo-González T., Larralde C. (2004). Host gender in parasitic infections of mammals: An evaluation of the female host supremacy paradigm. J. Parasitol..

[12] Meneses G., Berzunza M., Becker I., Bobes R.J., Rosas G., Sciutto E., Fragoso G. (2009). Taenia crassiceps cysticercosis: Variations in its parasite growth permissiveness that encounter with local immune features in BALB/c substrains. Exp. Parasitol..

[13] Larralde C., Morales J., Terrazas I., Govezensky T., Romano M. (1995). Sex hormone changes induced by the parasite lead to feminization of the male host in murine *Taenia crassiceps* cysticercosis. J. Steroid Biochem. Mol. Biol..

[14] Terrazas L.I., Bojalil R., Govezensky T., Larralde C. (1998). Shift from an early protective Th1-type immune response to a late permissive Th2-type response in murine cysticercosis (*Taenia crassiceps*). J. Parasitol..

[15] I Terrazas L., Bojalil R., Govezensky T., Larralde C. (1994). A role for 17-beta-estradiol in immunoendocrine regulation of murine cysticercosis (*Taenia crassiceps*). J. Parasitol..

[16] Huerta L., Terrazas L.I., Sciutto E., Larralde C. (1992). Immunological Mediation of Gonadal Effects on Experimental Murine Cysticercosis Caused by *Taenia crassiceps* Metacestodes. J. Parasitol..

[17] McLennan D.A., Brooks D.R. (1991). Parasites and Sexual Selection: A Macroevolutionary Perspective. Q. Rev. Biol..

[18] Fragoso G., Meneses G., Sciutto E., Fleury A., Larralde C. (2008). Preferential Growth of *Taenia crassiceps* Cysticerci in Female Mice Holds Across Several Laboratory Mice Strains and Parasite Lines. J. Parasitol..

[19] Hernandez-Bello R., Nava-Castro K., Muniz-Hernandez S., Nava-Luna P., Trejo-Sánchez I., Tiempos-Guzman N., Mendoza-Rodriguez Y., Morales-Montor J. (2012). Beyond the reproductive effect of sex steroids: Their role during immunity to helminth parasite infections. Mini-Rev. Med. Chem..

[20] Escobedo G., Larralde C., Chavarria A., Cerbón M.A., Morales-Montor J. (2004). Molecular mechanisms involved in the differential effects of sex steroids on the reproduction and infectivity of *Taenia crassiceps*. J. Parasitol..

[21] Escobedo G., Robert C.W., Carrero J.C., Morales-Montor J. (2005). Parasite regulation by host hormones: An old mechanism of host exploitation?. Trends Parasitol.

[22] Rankin L.C., Artis D. (2018). Beyond Host Defense: Emerging Functions of the Immune System in Regulating Complex Tissue Physiology. Cell.

[23] MacDonald A.S., Araujo M.I., Pearce E.J. (2002). Immunology of Parasitic Helminth Infections. Infect. Immun..

[24] Davidson R.A. (1985). Immunology of Parasitic Infections. Med. Clin. N. Am..

[25] Morales-Montor J., Larralde C. (2005). The role of sex steroids in the complex physiology of the host-parasite relationship: The case of the larval cestode of *Taenia crassiceps*. Parasitology.

[26] Dantzer R. (2018). Neuroimmune Interactions: From the Brain to the Immune System and Vice Versa. Physiol. Rev..

[27] McCorry L.K. (2007). Physiology of the autonomic nervous system. Am. J. Pharm. Educ..

[28] Ulloa L. (2013). The cholinergic anti-inflammatory pathway meets microRNA. Cell Res..

[29] Ulloa L. (2011). The Anti-Inflammatory Potential of Selective Cholinergic Agonists. Shock.

[30] Arteaga M., Chavarría A., Montor J.M. (2003). Immunoneuroendocrine communication network and homeostasis regulation: The use of hormones and neurohormones as immunotherapy. Rev. Investig. Clin. Organo Hosp. Enferm. Nutr..

[31] Valentin A.P., Kevin J.T. (2005). The cholinergic anti-inflammatory pathway. Brain Behav. Immun..

[32] Bottasso O., Morales-Montor J. (2009). Neuroimmunomodulation during Infectious Diseases: Mechanisms, Causes and Consequences for the Host. Neuroimmunomodulation.

[33] Pérez A.R., Bottasso O., Savino W. (2009). The Impact of Infectious Diseases upon Neuroendocrine Circuits. Neuroimmunomodulation.

[34] Jiang H., Chess L. (2009). Chapter 2 How the Immune System Achieves Self–Nonself Discrimination During Adaptive Immunity. Adv. Immunol..

[35] Segovia-Mendoza M., Morales-Montor J. (2019). Immune Tumor Microenvironment in Breast Cancer and the Participation of Estrogen and Its Receptors in Cancer Physiopathology. Front. Immunol..

[36] Kerage D., Sloan E.K., Mattarollo S.R., McCombe P.A. (2019). Interaction of neurotransmitters and neurochemicals with lymphocytes. J. Neuroimmunol..

[37] Arreola R., Villanueva L.E.B., Cruz-Fuentes C., Velasco-Velazquez M.A., Garcés-Alvarez M.E., Hurtado-Alvarado G., Quintero-Fabian S., Pavón L. (2015). Immunomodulatory Effects Mediated by Serotonin. J. Immunol. Res..

[38] Kumar A., Russell R.M., Pifer R., Menezes-Garcia Z., Cuesta S., Narayanan S., MacMillan J.B., Sperandio V. (2020). The Serotonin Neurotransmitter Modulates Virulence of Enteric Pathogens. Cell Host Microbe.

[39] Alonso-Trujillo J., Rivera-Montoya I., Rodriguez-Sosa M., Terrazas L.I. (2007). Nitric oxide contributes to host resistance against experimental *Taenia crassiceps* cysticercosis. Parasitol. Res..

[40] Fuchs B.A., Campbell K.S., Munson A.E. (1988). Norepinephrine and serotonin content of the murine spleen: Its relationship to lymphocyte beta-adrenergic receptor density and the humoral immune response in vivo and in vitro. Cell. Immunol..

[41] Grailer J., Haggadone M., Ward P. (2013). Catecholamines promote an M2 macrophage activation phenotype (P1299). J. Immunol..

[42] Murray K., Godinez D.R., Brust-Mascher I., Miller E.N., Gareau M.G., Reardon C. (2017). Neuroanatomy of the spleen: Mapping the relationship between sympathetic neurons and lymphocytes. PLoS ONE.

[43] Herr N., Bode C., Duerschmied D. (2017). The Effects of Serotonin in Immune Cells. Front. Cardiovasc. Med..

[44] Ishikawa N., Goyal P.K., Mahida Y.R., Li K.F., Wakelin D. (1998). Early cytokine responses during intestinal parasitic infections. Immunology.

[45] Hewitson J.P., Grainger J.R., Maizels R.M. (2009). Helminth immunoregulation: The role of parasite secreted proteins in modulating host immunity. Mol. Biochem. Parasitol..

[46] Martínez-Saucedo D., Ruiz-Rosado J.D.D., Terrazas C., Callejas B.E., Satoskar A.R., Partida-Sánchez S., Terrazas L.I. (2019). Taenia crassiceps-Excreted/Secreted Products Induce a Defined MicroRNA Profile that Modulates Inflammatory Properties of Macrophages. J. Immunol. Res..

[47] Patel N., Kreider T., Urban J.F., Gause W.C. (2009). Characterisation of effector mechanisms at the host:parasite interface during the immune response to tissue-dwelling intestinal nematode parasites. Int. J. Parasitol..

[48] Henry E.K., Inclan-Rico J.M., Siracusa M.C. (2017). Type 2 Cytokine Responses: Regulating Immunity to Helminth Parasites and Allergic Inflammation. Curr. Pharmacol. Rep..

[49] Smith K.A., Maizels R.M. (2014). IL-6 controls susceptibility to helminth infection by impeding Th2 responsiveness and altering the Treg phenotype in vivo. Eur. J. Immunol..

[50] Roberts C.W., Walker W., Alexander J. (2001). Sex-Associated Hormones and Immunity to Protozoan Parasites. Clin. Microbiol. Rev..

[51] Klein S.L. (2004). Hormonal and immunological mechanisms mediating sex differences in parasite infection. Parasite Immunol..

[52] Miller C.M.D., Smith N.C., Ikin R.J., Boulter N.R., Dalton J.P., Donnelly S. (2009). Immunological Interactions between 2 Common Pathogens, Th1-Inducing Protozoan *Toxoplasma gondii* and the Th2-Inducing Helminth *Fasciola hepatica*. PLoS ONE.

[53] Pearce E.J., Kane C.M., Sun J., Taylor J.J., McKee A.S., Cervi L. (2004). Th2 response polarization during infection with the helminth parasite Schistosoma mansoni. Immunol. Rev..

[54] Moreau E., Chauvin A. (2010). Immunity against Helminths: Interactions with the Host and the Intercurrent Infections. J. Biomed. Biotechnol..

[55] Che Q., Liu B., Liao Y., Zhang H., Yang T. (2014). Activation of a positive feedback loop involving Il 6 and aromatase promotes intratumoral 17Ò estradiol biosynthesis in endometrial carcinoma microenvironment. Cancer Cell Biol..

[56] Morales-Montor J., Baig S., Hallal-Calleros C., Damian R. (2002). Taenia crassiceps: Androgen reconstitution of the host leads to protection during cysticercosis. Exp. Parasitol..

[57] Morales-Montor J., Baig S., Mitchell R., Deway K., Hallal-Calleros C., Damian R.T. (2001). Immunoendocrine interactions during chronic cysticercosis determine male mouse feminization: Role of IL-6. J. Immunol..

[58] Bechir M., Schelling E., Hamit M.A., Tanner M., Zinsstag J. (2011). Parasitic Infections, Anemia and Malnutrition Among Rural Settled and Mobile Pastoralist Mothers and Their Children in Chad. EcoHealth.

[59] Carpio A., Romo M., Parkhouse R.M.E., Short B., Dua T. (2016). Parasitic diseases of the central nervous system: Lessons for clinicians and policy makers. Expert Rev. Neurother..

[60] Dorais F.J., Esch G.W. (1969). Growth rate of two Taenia crassiceps strains. Exp. Parasitol..

